# A Non-Invasive Tool for Real-Time Measurement of Sulfate in Living Cells

**DOI:** 10.3390/ijms21072572

**Published:** 2020-04-07

**Authors:** Urooj Fatima, Mohammad K. Okla, Mohd Mohsin, Ruphi Naz, Walid Soufan, Abdullah A. Al-Ghamdi, Altaf Ahmad

**Affiliations:** 1 Department of Botany, Faculty of Life Sciences, Aligarh Muslim University, Aligarh 202001, India; uroojf15@gmail.com (U.F.); ruphinaz@gmail.com (R.N.); 2 Botany and Microbiology Department, College of Science, King Saud University, P.O. Box. 2460, Riyadh 11451, Saudi Arabia; abdaalghamdi@ksu.edu.sa; 3 Department of Biosciences, Jamia Millia Islamia, New Delhi 110025, India; mmohsin1@jmi.ac.in; 4 Plant Production Department, Faculty of Food and Agricultural Sciences, King Saud University, P.O. Box 2460, Riyadh 11451, Saudi Arabia; wsoufan@ksu.edu.sa

**Keywords:** sulfate, FRET, fluxomics

## Abstract

Sulfur (S) is an essential element for all forms of life. It is involved in numerous essential processes because S is considered as the primary source of one of the essential amino acids, methionine, which plays an important role in biological events. For the control and regulation of sulfate in a metabolic network through fluxomics, a non-invasive tool is highly desirable that opens the door to monitor the level of the sulfate in real time and space in living cells without fractionation of the cells or tissue. Here, we engineered a FRET (fluorescence resonance energy transfer) based sensor for sulfate, which is genetically-encoded and named as FLIP-SP (Fluorescent indicator protein for sulfate). The FLIP-SP can measure the level of the sulfate in live cells. This sensor was constructed by the fusion of fluorescent proteins at the N- and C-terminus of sulfate binding protein (sbp). The FLIP-SP is highly specific to sulfate, and showed pH stability. Real-time monitoring of the level of sulfate in prokaryotic and eukaryotic cells showed sensor bio-compatibility with living cells. We expect that this sulfate sensor offers a valuable strategy in the understanding of the regulation of the flux of sulfate in the metabolic network.

## 1. Introduction

Sulfur (S) has assigned an important significance for all life forms. It is an essential nutrient for growth and development. Plants constitutively require sulfur for the biosynthesis of sulfur-containing amino acids (methionine, cysteine), vitamins (biotin and thiamine), secondary metabolites, prosthetic groups, glutathione, and a range of coenzymes. The sulfur is taken up and transported to shoots in the form of inorganic sulfate (SO_4_^2−^) from the rhizosphere by roots. Incorporation of sulfate into cysteine is regarded as the biotic aspect of the natural sulfur cycle [1]. Human and animals fulfill the demand of sulfur by plants, algae, fungi, yeast, and many prokaryotes, which can take up the sulfate, reduce, and assimilate it. Glucosinolates, sulfur-containing compounds found in the *Brassicaceae* family of plants, are essential for plant defense against various herbivores and pathogens. Cruciferous family containing sulfur compounds are responsible for fighting against bacteria, *Helicobacter pylori*, which causes stomach cancer [2]. Methionine, a sulfur-containing amino acid, is crucial for the initiation of protein synthesis. Thus, deficiency in sulfur slows down or hinders the process of translation initiation [3]. In *Arabidopsis thaliana*, S deprivation led to transcriptomic changes that involve about approximately 1500 genes [4,5]. The crucial role of sulfate in the growth and yield of crops has attracted researchers to work on its assimilation, uptake, and transport mechanism. Despite the importance of sulfate, a thorough understanding of intracellular regulation of sulfate homeostasis is limited because of the inability to assess intracellular sulfate concentrations with high spatial and temporal resolution in living tissue. To understand the control and regulation of metabolic networks, we need to know the flow of metabolites through the metabolic pathway in time and space. Fluxomics provides quantitative information on the dynamics of ions and metabolites at sub-cellular and cellular compartmentalization by measurement of metabolome wide fluxes. To study the flux of sulfate, a tool is needed to determine the concentration of sulfate in a physiological environment. A tool that may help to visualize how the concentration of sulfate varies in a biological system, which can be a tissue or cell and that can detect how sulfate level changes in response to environmental stimuli. In previous research, various spectrometry techniques have been used that allowed for piloting of the biochemical properties of the living cell, but they have limited temporal and spatial resolution. The FRET (fluorescence resonance energy transfer) based genetically-encoded nanosensor has been proven to be a powerful tool that allows for the measurement of the metabolites at subcellular level in living cells and in a non-destructive manner [6,7,8,9].

The nanosensor consists of bacterial periplasmic binding proteins as a ligand sensing domain sandwiched between a FRET pair of fluorescent proteins [10]. In our study, we generated a FLIP-SP nanosensor by sandwiching sulfate binding protein (sbp) of *Salmonella typhimurium* between enhanced cyan fluorescent protein (ECFP) as a donor and mVenus as an acceptor, expressed in CodonPlus strain of *E. coli.* The FRET is measured as the ratio of the acceptor emission intensity and the donor emission intensity by exposing a sample to the donor excitation light in a microplate reader. This study demonstrates that FLIP-SP nanosensors can be used to monitor the level of sulfate in living cells.

## 2. Results

### 2.1. Designing and Engineering of the FRET-Based Sulfate Sensor

**Figure 1:**
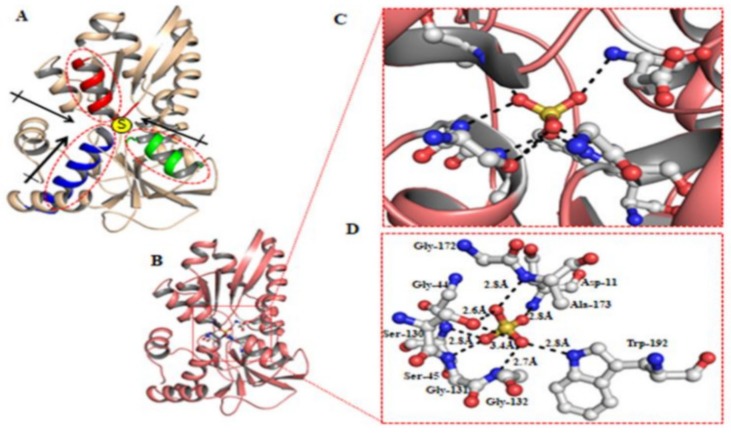
Computational analysis. Molecular docking of sulfate binding protein from *Salmonella typhimurium.* (**A**) Depiction of three α-helices form. (**B**) The oxygen atom of sulfate ion offers seven hydrogen bonds to amino acid residues of sbp. (**C**) Enlarge the view of sulfate ion offers seven hydrogen bonds to amino acid residues. (**D**) Two hydrogen bonds to Ser130 (2.6 and 3.4Å) of sbp, one with Ala173 (2.8 Å), one with Asp11 (2.8 Å), one with Ser45 (2.8 Å), one hydrogen bonds with Trp192 (2.8 Å), one bond with Gly131 (2.7 Å), and one bond with Gly132 (2.7 Å).

Sulfate-binding protein (sbp) has been identified as a periplasmic binding protein of *Salmonella typhimurium.* It plays an essential role in high specificity and high-affinity transporters. Protein data bank (RCSB-PDB) was used to retrieve the crystal structure of sbp (PDBID-5UM2, resolution 1.14 A). In silico molecular docking analysis revealed the intermolecular distance between the interacting residues of sbp–sulfate complexes. Sulfate binding protein (sbp) interacts with sulfate ions under the α-helix ‘macrodipole model’ [11]. It has been found that these macrodipoles help to provide an enormous number of electrostatic interactions to the complex. In case of the sbp, the three α-helices form three macrodipoles, as shown in Figure 1A. Based on this model, it has been proposed that the N-terminal and C-terminal of each helix bear half negative and half positive charges separated by the length of a helix. Furthermore, in the case of sbp, it has been found that the C-terminal of these helices was capped by positively charged arginine residues. These arginine residues compensate for the macrodipoles’ partial negative charges and transform them into large monopoles with a greater ability to attract the negatively charged ligands like sulfate ions. Results showed that oxygen atoms of the sulfate ion offer seven hydrogen bonds to the amino acid residues of sbp (Figure 1B,C). The oxygen atoms of sulfate offer two hydrogen bonds to Ser130 (2.6 and 3.4Å) of sbp, one with Ala173 (2.8 Å), one with Asp11 (2.8 Å), one with Ser45 (2.8 Å), one hydrogen bonds with Trp192 (2.8 Å), one bond with Gly131 (2.7 Å), and one bond with Gly132 (2.7 Å) (Figure 1D). These observations suggest that the sulfate ion bears high affinity toward sbp, and the sbp–sulfate complex is stabilized by an ample number of interactions. 

**Figure 2:**
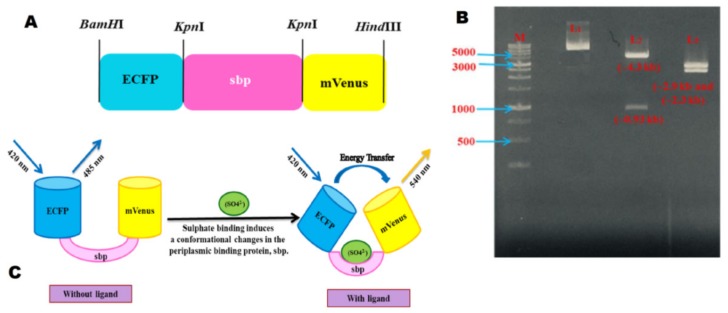
Schematic representation of the design of the sulfate nanosensor. (**A**) Design of restriction sites of ECFP, sbp, and mVenus; (**B**) Restriction digestion of the cloned ECFP_sbp_mVenus construct in the pRSET-B vector. M = DNA ladder, L1 = uncut plasmid, L2 = digested product with *Kpn*I, L3 = digested product with *Bam*HI and *Hin*dIII; (**C**) Schematic representation of working of the sulfate nanosensor. Upon binding of sulfate to sbp, ECFP and mVenus come closure, and the FRET occurs.

Nucleotide sequences were retrieved from the KEGG database. A design of the nanosensor is given in Figure 2. Figure 2A illustrates the proper alignment of module genes with a frame-up of restriction sites to assemble all the components of the sulfate nanosensor. To engineer the nanosensor, the ECFP_sbp_mVenus construct was cloned in the pGEM-Teasy vector to develop pGEM-T_ECFP_sbp_mVenus. Later, the ECFP_sbp_mVenus was excised by using *Bam*HI and *Hin*dIII restriction endonuclease enzymes. The liberated ECFP_sbp_mVenus construct was then ligated with the pRSET-B vector at *Bam*HI and *Hin*dIII sites.

The confirmation of the chimeric construct was done by restriction digestion with the *Kpn*I restriction enzyme released sbp gene from the whole construct and generated two bands (~4.3 Kb and ~0.93 Kb). The construct was further digested by *Bam*HI and *Hin*dIII to the release ECFP_sbp_mVenus construct separating the pRSET-B vector, giving two bands of size ~2.3 kb and ~2.9 kb, respectively (Figure 2B). The resultant product is the sulfate nanosensor. It was further verified by the full-length Sanger sequencing to check the fidelity, and was named as the FLIP-SP nanosensor. In the presence of the sulfate molecule, the conformational changes occurred in the sbp, which resulted in a steady pairing of ECFP and mVenus for the quantification of sulfate in real-time. The sbp was used as a sensing domain. Figure 2C illustrates the chimeric construct pRSET-B_ECFP-sbp-mVenus, demonstrating that no energy transfers in the unliganded state, while in a liganded state, energy is transferred. Upon sulfate binding, the conformational change occurs in the sulfate binding periplasmic protein, which causes the ECFP–mVenus fluorescent protein pair to come into proximity and in parallel alignment and also change the distance (˂10 nm) between the two fluorescent proteins, which fulfill the conditions of FRET to occur.

### 2.2. Purification and In Vitro Assay of the Sulfate Sensor

**Figure 3:**
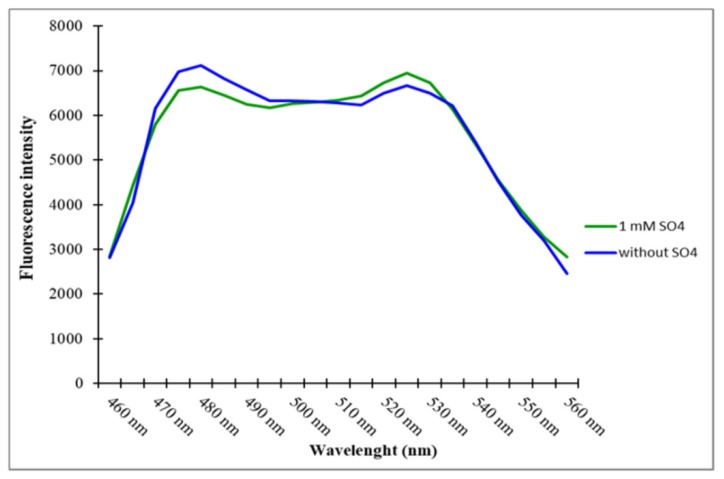
Spectral analysis of the sensor. Emission intensity was recorded in the absence and presence of 1 mM of sulfate after excitation of 435 nm.

To determine the in vitro analysis of the sulfate sensor, it was transformed and expressed by inducing with 0.5 mM of Isopropyl β-D-1-thiogalactopyranoside (IPTG) to obtain a high yield in BL21-CodonPlus (DE3) strain of *E. coli*. These fluorescent protein indicators ECFP and mVenus are quite sensitive to photobleaching. Therefore, maintenance of dark conditions fraternized the photostability and also improved the proper folding efficiency. The expressed bacterial cells were pelleted down by centrifugation, and the pellet was resuspended in the Tris-Cl buffer (pH 7.5), followed by lysis using ultrasonication (Sonics, Newtown, CT, USA). The bacterial lysates was centrifuged again for 30 min, and the sensor proteins were purified from using Ni-NTA resin. Emission spectral analysis of the sensor showed the shift in the emission spectra of ECFP and mVenus by excitation at 435 nm in the presence of sulfate. In the absence of sulfate, no shift was observed in the emission spectrum. The sharp shift in emission spectrum was observed with the addition of 1 mM sulfate, which triggered a decrease in the fluorescence emission intensity of ECFP and an increase in the fluorescence emission intensity of mVenus. These shifts in the spectrum demonstrated that the addition of 1 mM of sulfate led to the conformational changes in sbp, which brought the two fluorophores, ECFP and mVenus, into close proximity (i.e., <10 nm in distance), and in parallel alignment, which transferred energy non-radiatively from the donor (ECFP) to acceptor fluorophore (mVenus), and thus increased the fluorescence emission intensity of mVenus (Figure 3). The FLIP-SP sensor accomplished this specification as there were conformational changes to PBPs and a shift in the emission spectra after the addition of 1 mM of sulfate.

**Figure 4:**
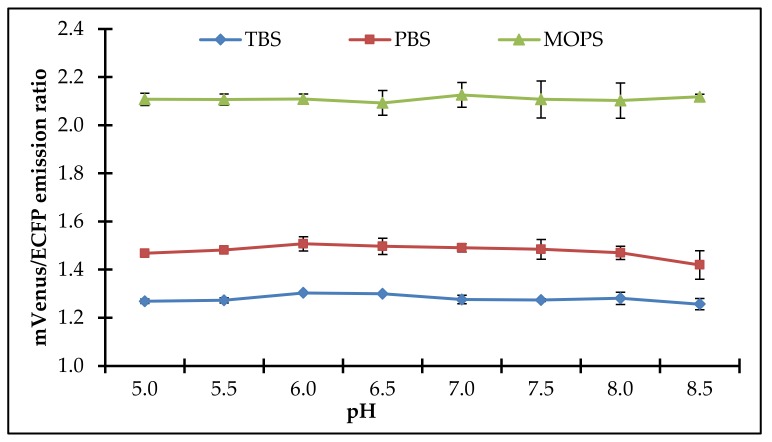
FLIP-SP sensor sensitivity toward pH with various buffers. Values are the mean of three replicates. Vertical bars show the standard deviation.

The kinetics of the FLIP-SP sensor protein under various buffers system i.e., Tris-buffered saline (TBS), Phosphate buffered saline (PBS), and 3-(*N*-morpholino)propanesulfonic acid (MOPS) at diverse pH ranging from 5.0 to 8.5 were investigated. We reported that the FLIP-SP sensor in MOPS buffer significantly showed the maximum stability and efficiency while the least stability and efficiency were observed in TBS and PBS buffers. The better performance in MOPS buffer is possible because MOPS lacks the ability to form a complex with most metal ions and is recommended for use as a non-coordinating buffer in solutions with metal ions. Therefore, we chose 20 mM of MOPS buffer with a pH = 6.5 for further experimental study. To examine the pH stability of the FLIP-SP sensor protein, the mVenus/ECFP emission ratio was recorded in a series of pH conditions ranging from 5.0 to 8.5. The sulfate binding affinity was unaffected within these pH ranges as no changes occurred in the FRET ratio (Figure 4). The outcome of the pH stability of sensor protein implies that the FLIP-SP sensor is very acceptable and has the potential to examine the flux rate of sulfate at different pH in both in vitro and in vivo in real-time. 

**Figure 5:**
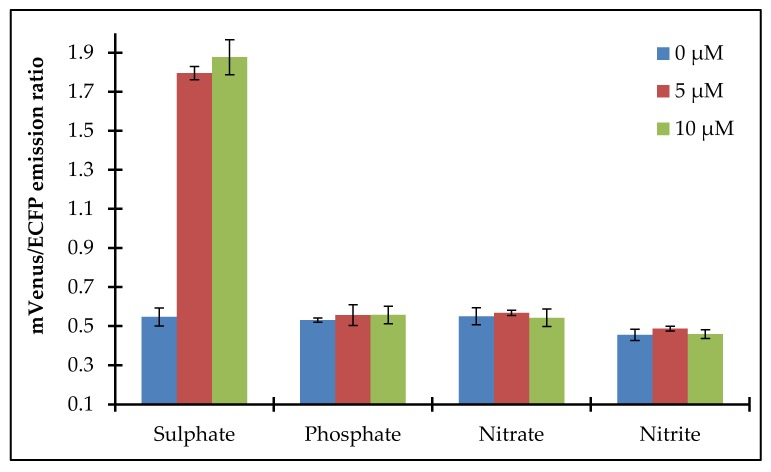
Specificity of the FLIP-SP sensor with different molecules. Error bars show the standard deviation of the three independent replicates.

Testing the in vitro specificity of the FLIP-SP nanosensor is an important feature in which to characterize the nanosensor. Specificity analysis of the FLIP-SP was performed on a microplate reader with different molecules, sulfate, phosphate, nitrate, and nitrite at different concentrations in a set of triplicates. In the absence of sulfate (control) and in the presence of other related molecules, consequential changes in emission intensity ratio were not reported by the FLIP-SP nanosensor. However, with the addition of 5 µM and 10 µM sulfate, consequential changes in emission intensity ratio (mVenus em/ECFP em) were scanned by the FLIP-SP nanosensor, indicating that this sensor response is specific to sulfate only. The FLIP-SP nanosensor was found to be highly specific at 10 µM of sulfate (i.e., this FLIP-SP nanosensor gives a quick and instant response by the addition of 10 µM of sulfate (Figure 5). 

### 2.3. Affinity and Mutants of Sulfate Sensor

**Figure 6:**
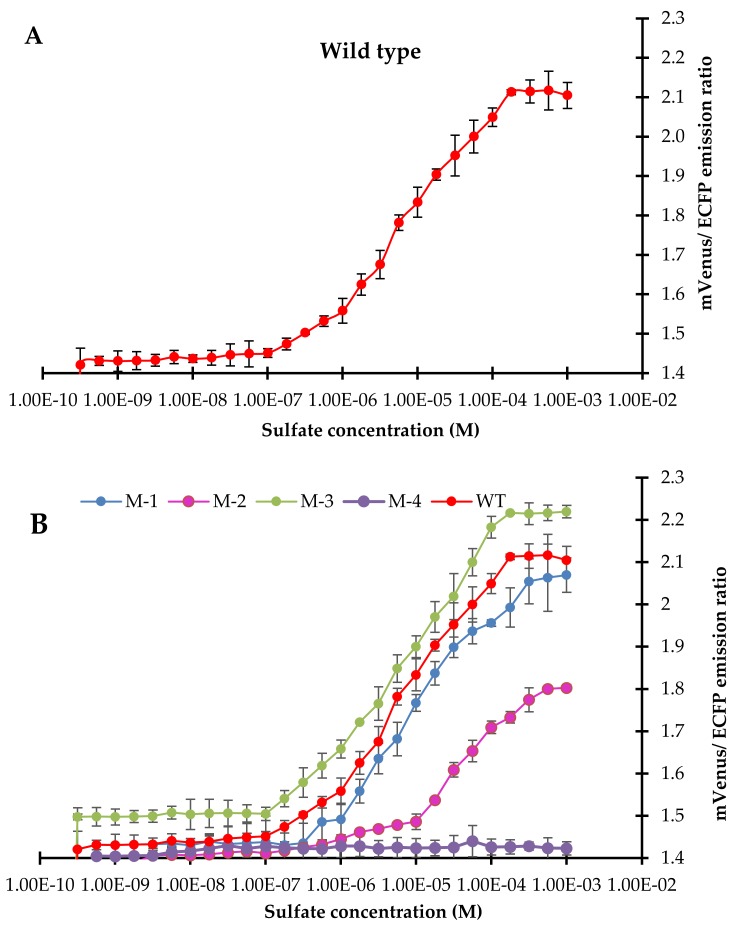
Titration analysis of the sensor protein. (**A**) In vitro emission ratio measurement by the FLIP-SP nanosensor. (**B**) In vitro ligand-dependent mVenus/ECFP emission ratio change of wild type (WT) and mutant sensors in the presence of different sulfate concentrations. Values are the means of three independent replicates. Vertical bars show the standard deviation.

For affinity measurement (*K*_d_), the FLIP-SP nanosensor was determined by measuring the emission intensity ratio in a microplate reader holding a 96-well black plate with each well containing a sample in a set of triplicates. Titration of the expressed sensor protein was carried out with the different concentrations of sulfate to investigate the FRET ratio change at diverse concentrations ranging from nM to mM, in order to obtain a sigmoidal curve that displayed the significant change in the mVenus/ECFP emission ratio that started at 100 nM and saturated at a 100 µM sulfate concentration (Figure 6A). The measured affinity (*K*_d_) of 10 µM was obtained with the sulfate for the FLIP-SP nanosensor. A total of four mutants (FLIP-11, FLIP-130, FLIP-132, and FLIP-192) of FLIP-SP were also generated by creating point mutation in the wild type of FLIP-SP by using site-directed mutagenesis in which residues (aspartate, serine, glycine, and tryptophan) were replaced by glycine, isoleucine glutamine, and alanine, respectively. With the basic ideas, residues were converted into hydrophobic or polar amino acid residues. The increased hydrophobicity by hydrophobic amino acids and hydrogen bond formation by polar amino acid residues provided the conformational stability of the proteins. In vitro, ligand-dependent FRET ratio change of all four mutants of FLIP-SP in the presence of sulfate was recorded the same as for wild type sensors (Figure 6B). The calculated *K*_d_ of WT and mutants FLIP-11, FLIP-192, FLIP130, and FLIP132 were 10 µM, 0 µM (no binding), 6 µM, 8 µM, and 45 µM, respectively (Table 1). 

The generated mutants had different *K*_d_ compared to those of the WT. Generation of a point mutation in the binding pocket residue alters the substrate binding properties of the sulfate sensor and can enhance the detection range of the sulfate sensor. Among all the mutants of the sensors studied, the FLIP-192 sensor showed the maximum FRET ratio changes of 2.2 and had a binding constant (*K*_d_) of 6 μM and thus, considered the best for real-time monitoring of the flux of sulfate dynamics in living cells. Absolute concentration of the sulfate can be calculated using the standard curve between the FRET ratio and the concentration of sulfate (Figure S1).

### 2.4. Measurement of Flux of Sulfate Uptake in Living Cells

#### 2.4.1. In Bacteria

**Figure 7:**
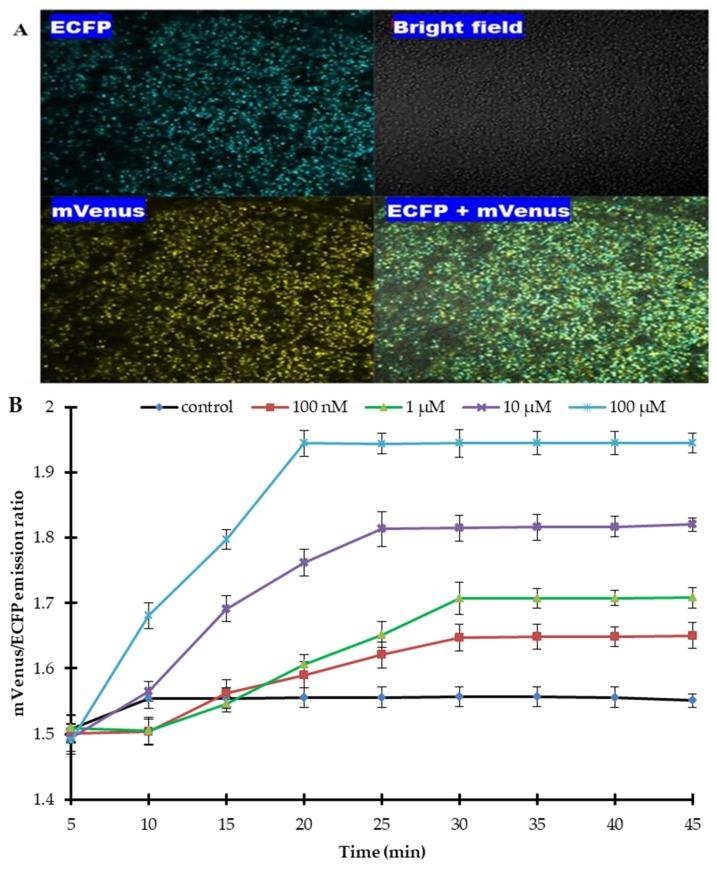
Real-time monitoring of sulfate levels in *E. coli* using the FLIP-SP nanosensor. (**A**) Confocal imaging of *E. coli* BL21-CodonPlus (DE3) cells expressing the FLIP-SP. ECFP, mVenus, and ECFP + mVenus (merged) indicate the specific excitation and emission wavelength of the fluorophores. (**B**) Measurement of the sulfate uptake for 45 min. in *E. coli* as the Venus/ECFP emission ratio changes in response to different concentrations of sulfate. Values are the mean of three independent replicates. Vertical bars indicate standard errors.

To monitor the flux rate of sulfate in real-time and its localization in live bacterial cells, the FLIP-SP nanosensor was transformed and expressed into the cytosol of the BL21-codon plus strain of *E. coli*. Confocal images of the live *E. coli* BL21-codon plus (DE3) cells were acquired by using a confocal laser scanning microscope (CLSM), which showed that the live bacterial cells were successfully expressed by the FLIP-SP nanosensor (Figure 7A). For in vivo bacterial assay, the suspension of the expressed bacterial cells was poured into a 96-well microplate reader without sulfate and with different concentrations of sulfate. The change in the emission ratio of mVenus/ECFP was monitored for a total length of 45 min, imposing the emission ratio change at an interval of every 5 min. Without sulfate (control), saturation occurs after 5 min, showing that the bacterial responses to the minimal sulfate level already exists in itself. At 100 nM and 1 µM, there is a delay before the emission ratio starts to increase. This could be because at these lower concentrations, sulfate enters slowly inside the cell, and hence a delayed response was observed by a sensor to sense sulfate. At a higher level of sulfate, the concentration of sulfate is large enough to enter fast inside the cell. At 100 nM and 1 µM, a saturation of FRET ratio was observed at 30 min of incubation in the live *E. coli* BL21-codon plus cells. At 10 µM of sulfate concentration, the saturation of the FRET ratio was observed after 25 min. The fastest saturation (i.e., maximum accumulation of sulfate was observed at 100 µM concentration, i.e., after 20 min). Figure 7B illustrates that at a higher concentration of sulfate (100 µM), the acquired time will be minimized by 20 min, (i.e., at a higher concentration, the time of accumulation will be less and vice versa). All the experiments were done in triplicate to obtain a standard deviation to ensure that our sample gave the same result each time we tested. The in vivo results demonstrate that the FLIP-SP sensor responded to the addition of sulfate at a different concentration, which reveals the configuration of the FLIP-SP sensor to study the flux rate of sulfate dynamics in living cells at an actual time during which a biological process takes place.

#### 2.4.2. In Yeast

**Figure 8:**
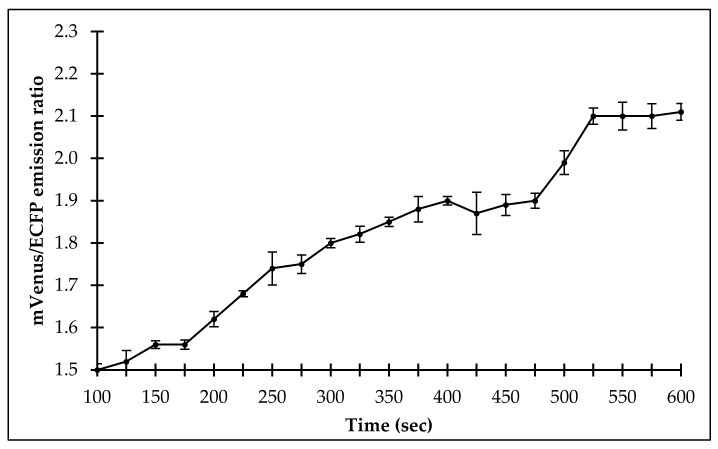
Real-time monitoring of sulfate levels in yeast using the FLIP-SP nanosensor. Level of sulfate in yeast cells was monitored by supplying 10 µM of sulfate for 600 s. Values are the mean of three independent replicates. Vertical bars indicate standard errors.

In vivo sulfate uptake was monitored for 600 s. The addition of 10 µM sulfate with the FLIP-SP expressing yeast cells showed the changes in the fluorescence emission intensity ratio of mVenus/ECFP. It was observed that there was an increase in the FRET ratio until 525 s, meaning that yeast cells are gradually uptaking sulfate, and after that, saturation started up to 600 s. This result indicates that sulfate was transported into the cytosol, and the flux rate was also steadily monitored in the eukaryotic system (Figure 8).

#### 2.4.3. In Plant

**Figure 9:**
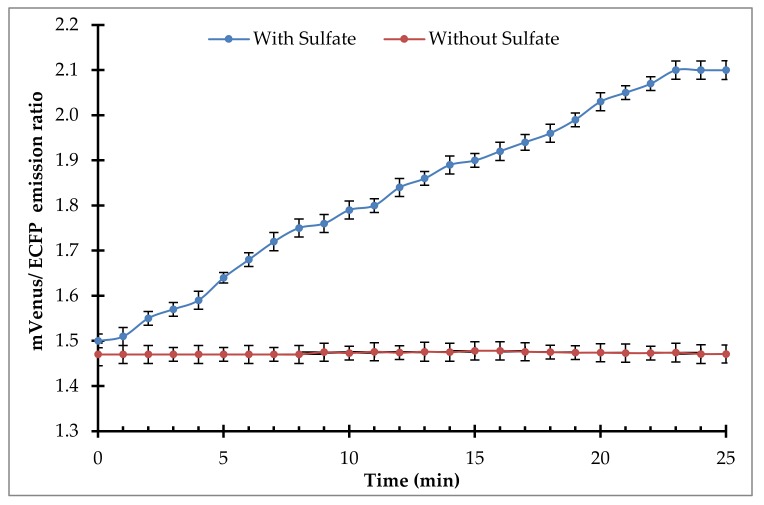
Real-time monitoring of level of sulfate using the FLIP-SP nanosensor in the transformed *Arabidopsis thaliana* cells in the absence and presence of sulfate.

*Arabidopsis thaliana* cells, transfected with the pEarleyDate100_ECFP_sbp_mVenus construct, showed that the sensor protein was expressed in the cytosol of the plant cells. A persistent FRET ratio was observed in the absence of sulfate, but the slow and consistent addition of sulfate to the suspension culture of plant cells increased the FRET ratio from 1.5 to 2.1, saturating at 23 min of incubation (Figure 9). This study indicates that the sulfate was transported in the cytosol of the eukaryotic cells and that the FLIP-SP sensor tool has the potential to study the real-time monitoring of sulfate flux in a metabolic network and to study the sulfate use efficiency in a metabolic pathway.

## 3. Discussion

In this study, we successfully engineered the first FRET-based genetically-encoded nanosensor for sulfate, known as “FLIP-SP”, to monitor the level of sulfate in real-time in live cells. The traces of sulfate in each cellular and sub-cellular compartment are highly dynamic in live plants. Therefore, it is complex to understand the mechanism for the regulation of sulfate homeostasis. This nanosensor paves the way to monitor the rate of sulfate uptake in living cells without disruption of the cells. One can utilize this FLIP-SP nanosensor as a non-invasive unique tool to detect a single intermediate, or to find the regulatory switch in their metabolic pathway, which enables researchers to study the intracellular level of sulfate in living cells with appropriate temporal and spatial resolution. This nanoscale gizmo offers high advantages over conventional analytical methods. Molecular docking studies of sulfate were performed by using AutoDock Vina and the AutoDock 4 package [12]. Atomic coordinates of sulfate binding protein (sbp) of *Salmonella typhirnurium* were taken from the Protein Data Bank (www.rcsb.org), PDB ID 1SBP [13]. Further procedures were followed as per the previously published protocol [14,15]. Finally, the complexes were visualized by PyMOL. Molecular docking was used to see the interactions between sulfate ions and amino acid residues of sbp. Furthermore, it was noticed that the charge-dipole interactions were the major determinants in the sbp–sulfate complex [13]. These interactions are of considerable importance for the rapid movement and active transport of ions [11]. Therefore, these transient movements are of utmost importance and make sbp a suitable bio-macromolecules for FRET-based studies with better FRET efficiency. A periplasmic binding protein (PBP), sbp from the bacteria, *Salmonella typhimurium*, and a pair of green fluorescent protein (GFP) variants were employed for the construction of FLIP-SP nanosensors. The PBPs are non-enzymatic bacterial receptors that undergo conformational changes upon sensing to a specific molecule and allow them to transport into the cell. The binding of a molecule to PBPs induced “Venus Flytrap” like conformational changes in PBP, which led the pair of fluorescent proteins (ECFP and mVenus) to come in parallel alignment and to a distance of <10 nm, which upon ligand binding will ultimately fulfill the requirements of FRET to occur [16]. Bacteria possess a diverse range of PBPs with the specificity of different molecules. The crystal structure of many of the well-characterized PBPs is available in an online database, which aids in designing the FRET-based genetically encoded nanosensor with a different pair of a variant of GFP at the N- and C- terminus of PBP for monitoring. Imaging various substrates in vitro and in vivo that translate into significant FRET changes upon binding of specific analytes to PBP. To engineer the FLIP-SP nanosensor for sulfate, sbp (a PBP) was utilized, which was tagged with ECFP and mVenus at its N- and C- terminus, respectively. In the previous research, it was documented that other transporters of sulfate have been reported to be responsible for nitrogen fixation in a symbiotic bacteria, *R. leguminosarum* [17,18]. Among the largest transporter gene family (i.e., ABC transporter), a SbpCysUWA is one of the known active systems for sulfate transport that consists of sbp. The sbp helps in ion uptake. The two permeases CysU and CysW (heterodimer) form a pore for substrate passage, and the ATPase CysA (homodimer) gives energy to the process [19]. The FRET-based genetically encoded nanosensor works as an important agent to estimate the presence and concentrations of specific metabolites at the cellular and subcellular level to monitor intracellular processes and to find the regulatory point of a metabolic pathway in real-time and in live cells [20,21,22,23]. Its non-invasive behavior and negligible cytotoxicity accomplished gains in insight into the metabolite, sensing transport, and compartmentalization. The general sensor principle of metabolite-induced conformational changes to PBPs, which is specific to their substrate, was used to create a sulfate nanosensor to monitor the level of sulfate that increased in emission intensity upon sulfate binding [24]. The study of pH stability of the generated nanosensor has become important because it exposes the degradation kinetics and potential toward less sensitivity of fluorescent proteins to fluctuations in intracellular pH as these proteins are highly sensitive toward changing environmental factors like photobleaching, which can hinder or alter the specificity of the nanosensor [25,26]. The pH stability experiments showed that these nanosensors are stable in a wide range of pH. This condition corresponds to the optimum physiological pH ranges of the majority of organisms. Therefore, these sensors are fit to apply on any type of cell. The strength of the specificity related to other molecules depends upon the sensory domain of the nanosensor that undergoes conformational changes by sensing and binding with the specific metabolites that cause a change in the FRET ratio. A similar approach has also been employed to measure the FRET ratio change of donor to acceptor emission intensity for monitoring the other nutrients and ions like phosphate [27], magnesium [28], and molybdenum [29]. Prior to estimating the equilibrium dissociation constant (*K*_d_) for various periplasmic binding proteins, equilibrium dialysis was used, but in our study, we determined an affinity based on the FRET ratio change and increase in fluorescence induced by conformational changes in the sensor protein. We expect that this study will help to develop many more FRET-based genetically encoded nanosensors based on steady-state fluorescence anisotropy. Up to now, a diverse range of FRET-based genetically encoded nanosensors have been designed and constructed by exploring different sets of fluorescent proteins and different ligand binding periplasmic proteins to acquire live-cell imaging of bacterial cells for the ideal representation of the nanosensors [30,31,32]. The FLIP-SP will be useful for studying sulfate uptake, translocation, and regulatory mechanisms controlling compartmental SO_4_^2−^ homeostasis. Analysis of the in vivo level of sulfate in the prokaryotic and eukaryotic system would increase our understanding in elucidating the regulatory point of a metabolic pathway, which helps to develop strategies for nutrient use efficiency of crop plants.

## 4. Materials and Methods 

### 4.1. Molecular Docking

Molecular docking was carried out using the AutoDock Vina and AutoDock 4 package. Atomic coordinates of the sulfate binding protein (SBP) of *Salmonella typhirnurium* were taken from the RCSB Protein Data Bank database, PDB ID 1SBP. Finally, the complexes were visualized by PyMOL. 

### 4.2. Designing and Construction of Sulfate Nanosensor

The ligand sensing domain of the sulfate nanosensor was sulfate-binding protein (sbp), refined at resolutions of 2.0 Å [33,34] and 1.7 Å [13]. The sbp gene was a procured form of *Salmonella typhimurium,* a Gram-negative bacteria, and is a primary periplasmic receptor of active transport. The sbp, a periplasmic binding protein, was placed between ECFP (donor fluorophore) and mVenus (acceptor fluorophore). Sequences of the ECFP and the mVenus were extracted from the pGWF1 vector. The Sbp sequence was retrieved from KEGG (Kyoto Encyclopedia Genomic Gene). The first 19 amino acids (signal peptide) and stop codon were detached from 5′ and 3′ ends, respectively, from the sbp gene. The signal peptide was detected by using the SignalP 4.1 server (CBS, Lyngby, Denmark). Polymerase chain reactions amplified all three genes (i.e., ECFP, mVenus, and sbp). Specifically, for the amplification of ECFP, mVenus, and sbp genes, Integrated DNA Technologies (IDT) was utilized for the design and synthesis of the primers. The ECFP was amplified by the forward primer with *Bam*HI and reverse primer with *Kpn*I (FP 5′-CGCggatccGTGAGCAAGGGCGAGGAGCT-3′, RP 5′-CGGggtaccCTTGTACAGCTCGTCCA TGC-3′); mVenus was amplified by the forward primer with *Kpn*I and reverse primer with *Hin*dIII (FP 5′-CGGggtaccGTGAGCAAGGGCGAGGAGCT-3′, RP 5′-CCCaagcttCTTGTACAGCTCGTCC ATGC-3′); and sbp was amplified by forward and reverse primers with *Kpn*I sites (FP 5′-CGGggtaccATTCAGTTACTTAACGTATCGTACG-3′, RP 5′-CGGggtaccTTTGCTGATTTGG TCGAACGTACCG-3′). Cloning of the individual ECFP, mVenus, and sbp genes was carried out in the pGEM^®^–T Easy vector (Promega, Madison, Wisconsin, USA). At first, mVenus was ligated to the ECFP–pGEM^®^–T Easy vector by restriction digestion, followed by ligation with the T4 DNA Ligase building ECFP–mVenus–pGEM^®^–T Easy vector cassette with appropriate restriction sites. The sbp gene with *Kpn*I restriction sites at both ends was inserted into the later cassette and yielded the chimeric construct of pGEM^®^–T_ECFP_sbp_mVenus. Sub-cloning of the ECFP_sbp_mVenus chimeric construct from pGEM^®^–T Easy to pRSET-B, a bacterial expression vector (Invitrogen, Carlsbad, CA, USA), was done by restriction digestion, followed by the ligation with pRSET-B, an expression vector containing His-tag. The His-tag purifies the chimeric protein via Ni-NTA chromatography. To check the fidelity, the plasmid of this chimeric construct were isolated and sequenced. The construct was named as FLIP-SP. The *E. coli* CodonPlus was transformed with pRSET-B-ECFP_sbp_mVenus for the expression of the sensor protein. Using Gateway cloning (Invitrogen, Carlsbad, CA, USA), the pYES-DEST52-ECFP_sbp_mVenus was created. The pYES-DEST52-ECFP_sbp_mVenus sequences were transformed into the *Saccharomyces cerevisiae*/URA3 strain BY4247 to express the sensor into an eukaryotic host (yeast). The sulfate sensor was expressed by a synthetic defined (SD) media, in which the cells were grown. The process took 3–5 days in which dextrose (2%) and galactose (1%) were added to provide a source of carbon and an inducer, respectively. 

### 4.3. Expression and Purification of Sulfate Nanosensor Protein

Sensor protein was expressed using the *E. coli* BL21-CodonPlus (DE3) cells. Purification of the expressed protein was carried out using Ni-NTA His-tag affinity chromatography (Novagen, Madison, WI, USA). 

### 4.4. Spectral Analysis, pH Stability, and Specificity of the Sulfate Sensor

Initially, purified sensor protein with Ni-NTA resin was diluted in Tris-Cl (20 mM, pH 7.5), making 0.25 mg per mL of protein. Spectral analysis of the diluted sample was carried out using a spectrofluorometer without adding the sulfate and after adding 1 mM sulfate. Excitation was done at 435 nm, and fluorescence emission intensity was recorded in the range of 460 nm to 560 nm. A plot of the emission intensities versus wavelength was obtained. For the pH stability of the sensor protein, the purified sensor protein was measured using phosphate buffer saline (PBS), Tris-buffer saline (TBS), and MOPS buffer (20 mM) in the pH range of 5.5–8.5. 

The interaction of sulfate with the sulfate sensor protein determines its specificity toward sulfate. To confirm the specificity of the sulfate sensor toward sulfate, the sensor protein was also exposed with other related molecules (phosphate, nitrate, and nitrite) by placing different concentrations of each molecule (0 µM, 5 µM, and 10 µM) in a microplate reader, and the emission intensity ratio (540 nm/485 nm) was recorded.

### 4.5. Affinity (K_d_) Analysis and Affinity Mutants of Sulfate Sensor

Titration of the sulfate sensor protein with different concentrations of sulfate was used to obtain a sigmoidal curve when a change in the FRET ratio (mVenus/ECFP) was plotted against the different concentrations of sulfate. The concentration of sulfate at which the change in FRET ratio becomes half of the maximum determines the affinity (*K*_d_) of the sulfate sensor protein. Theoretically, the *K*_d_ of the constructs are determined by fitting the ligand titration curve in a simple binding isotherm [30]. The affinity (*K*_d_) assay was carried out in a 96-well plate and monitored by using a multimode microplate reader. Each experiment was performed in three independent replicates. Based on the change in FRET ratio due to the non-radiatively transfer of energy, the results of affinity (*K*_d_) are described in the Results Section. To improve the physiological range of the genetically encoded nanosensor, to measure levels of sulfate at different physiological scales and to enhance the sulfate detection range, and affinity mutants (D11G, S130I, G132Q, W192A) were also created using the site-directed mutagenesis kit (Agilent, Santa Clara, CA, USA). The D11G, S130I, G132Q, and W192A were created by substituting aspartate^11^ with glycine, serine^130^ with isoleucine, glycine^132^ with glutamine, and tryptophan^192^ with alanine, respectively. Expression, purification, and analysis of the affinity of these mutant nanosensors of FLIP-SP were carried out using same procedure as that of wild type.

### 4.6. Measurement of Sulfate in Living Cells Using FLIP-SP

#### 4.6.1. In Bacteria

The ECFP_sbp_mVenus chimeric construct was ligated with the pRSET-B expression vector (Invitrogen, USA) and was transformed into the BL21-CodonPlus (DE3) strain of *E. coli.* A single transformed colony was grown on LB medium for three days at 20 °C in the dark to prevent fluorescent protein from photobleaching. Culture containing bacterial cells was induced by 0.5 mM isopropyl β-d-1-thiogalactopyranoside, and was grown until the OD_600_ reached 0.6. The cells were harvested, followed by pellet collection, and cells were resuspended in 20 mM MOPS buffer (pH 7.5). The FRET ratio (mVenus/ECFP emission) was measured in the 96 well microplate reader for 45 min at an interval of 5 min. Bacterial cell suspension (180 µL) was added to each well of the microplate. Various concentrations of sulfate (20 µL) were added to each well. One well was kept without sulfate (control). Imaging of the bacterial cells expressing the FLIP-SP sensor was carried out by using a confocal microscope.

#### 4.6.2. In Yeast

Monitoring of the sulfate flux in *Saccharomyces cerevisiae*/URA3 strain BY4247 using FLIP-SP was carried out as per the procedure of Mohsin et al. [30].

#### 4.6.3. In Plant

*Arabidopsis thaliana* was used as an experimental plant system for the characterization of the FLIP-SP sulfate sensor. The construct of ECFP_sbp_mVenus was shuttled to pEarleyGate100 (plant expression vector) through Gateway technology, generating pEarleyGate100_ECFP_sbp_mVenus. This construct was introduced in *Agrobacterium tumefaciens* strain EHA105. Disinfected seeds of *Arabidopsis* ecotype Col-0 were placed on a 0.5× Murashige and Skoog (MS) medium, supplemented with 2% sucrose, 0.7% agar, and 1 µM gibberellic acid, followed by incubation under controlled environmental conditions (28 ± 2 °C and 16 h light and 8 h dark period) until the appearance of true leaf. This young leaf explant of media was used to generate callus. Callus was generated on MS medium with 0.5 µM 2,4-D (auxin), 3% sucrose, and 0.62% agar (pH 5.8). The culture was kept at 25 ± 2 °C and 16/8 h light/dark period in a growth chamber.

*Agrobacterium* strain EHA105, harboring pEarleyGate100_ECFP_sbp_mVenus, was grown at 28 °C for 36 h in LB medium. Fifty mg/mL rifampicin and 50 mg/mL kanamycin were also added with the LB medium. Subsequently, the callus cells were transfected with the sulfate sensor construct (pEarleyGate100_ECFP_sbp_mVenus) through supplementation of the callus with the *Agrobacterium*. The culture was grown at 28 °C at 160 rpm until O.D._600_ reached 0.6. Drying of the explants on sterile filter paper and then plating on solid MS + 100 µM acetosyringone at 25 ± 2 °C for three days was also undertaken. Washing the explants was carried out by 500 mg/L cefotaxime. The explants were further washed with distilled water, and air-dried under laminar flow. Eventually, explants were placed on a co-cultivation medium augmented with basal MS salt, 250 mg/L cefotaxime, 10 mg/L BASTA, and 0.5 µM 2,4-D. Finally, cefotaxime and antibiotic were removed from the media and the cultures were incubated at 25 ± 2 °C for a 16 h photoperiod. Sulfate level in the suspension culture harboring the callus of the *Arabidopsis thaliana* containing pEarleyGate100_ECFP_sbp _mVenus was carried out under a Leica confocal microscope with a 10x objective. Ratio of the mVenus/ECFP emission intensities was recorded by using a 435/20 nm excitation filter and two emission filters (485/40 nm for ECFP and 535/30 nm for mVenus). LAS-AF software (Leica, Wetzlar, Germany) was used to display the FRET, mVenus/ECFP, and emission intensity ratio.

## 5. Conclusions

The sulfate binding protein (sbp) of *Salmonella typhimurium* along with the pair of fluorescent proteins (ECFP–sbp–mVenus) generated FRET-based genetically-encoded nanosensor (FLIP-SP). The nanosensor is capable of detecting the sulfate in vitro with high specificity and affinity to sulfate as well as being pH stable. The FLIP-SP functions efficiently in both the prokaryotic and eukaryotic systems. The level of sulfate can be analyzed in living cells without disruption of the tissue or cells, showing its non-invasiveness with high temporal and spatial resolution. We believe that this work represents the first report of a FRET-based genetically encoded nanosensor for sulfate measurement. This sensor would be useful to increase our understanding of elucidating the regulatory point of the metabolic pathway of sulfate uptake and assimilation, allowing the researchers and scientists to develop strategies for enhancing the nutritional value of crop plants to revolutionize agricultural, industrial, and therapeutic applications of sulfate. 

## Figures and Tables

**Table 1:** FLIP-SP wild type (WT) and mutants.

S.no	Sensor Name	Sequences	*K*_d_(µM)	Dynamic Range (µM)	∆Rmax §
1	FLIP-WT (WT)	Wild type	10	0.3 µM–90 µM	0.7
2	FLIP-130 (M-1)	S130I	8	0.2 µM–50 µM	0.6
3	FLIP-132 (M-2)	G132Q	45	40 µM–800 µM	0.4
4	FLIP-192 (M-3)	W192A	6	0.5 µM–80 µM	0.7
5	FLIP-11 (M-4)	D11G	no binding		

∆Rmax § is the maximal change in the FRET ratio of the sensor. ∆R_max_ = R_max_ − R_min_, where R_max_ and R_min_ correspond to the maximum and minimum FRET ratio.

## Supplementary Materials

Supplementary materials can be found at https://www.mdpi.com/1422-0067/21/7/2572/s1. Figure S1. Standard curve of sulfate concentration and FRET ratio.

## Abbreviations

FRET	Fluorescent Resonance Energy Transfer
ECFP	Enhanced Cyan Fluorescent Protein
sbp	Sulfate Binding Protein
