# Supplementary figures and images for: A Non-Invasive Tool for Real-Time Measurement of Sulfate in Living Cells
Source: Int J Mol Sci. 2020 Apr 7;21(7):2572. doi: 10.3390/ijms21072572 (PMC7177696; doi:10.3390/ijms21072572)

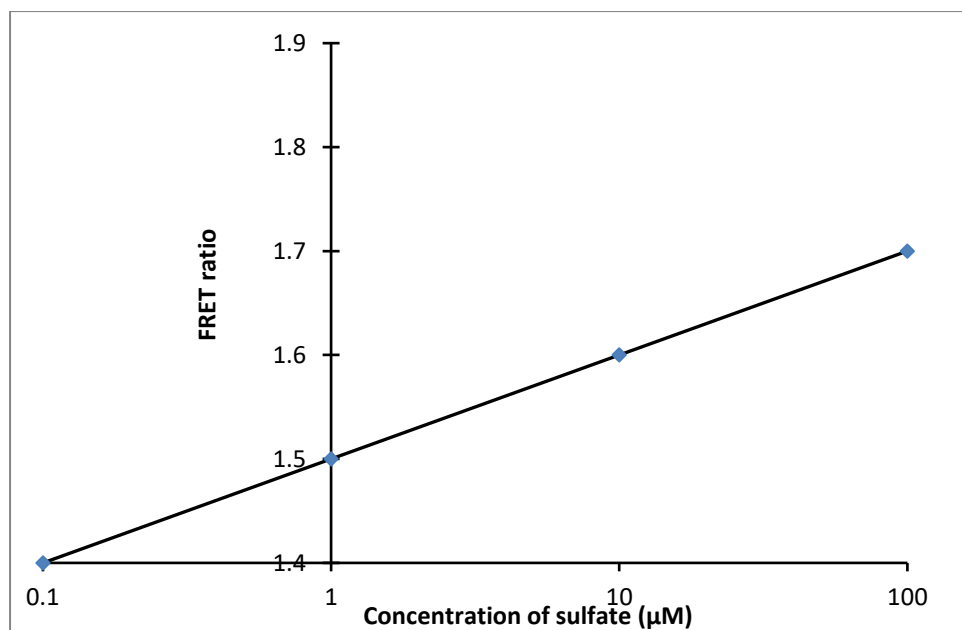

Figure S1. Standard curve of sulfate concentration and FRET ratio

Supplement: Supplementary file 1 [file ijms-21-02572-s001.pdf]
